# Comparative Analysis of PCI Strategies in Aortic Stenosis Patients Undergoing TAVI: A Systematic Review and Network Meta‐Analysis

**DOI:** 10.1002/clc.24324

**Published:** 2024-07-26

**Authors:** Parisa Fallahtafti, Hamidreza Soleimani, Pouya Ebrahimi, Amirhossein Ghaseminejad‐Raeini, Elaheh Karimi, Amirhossein Shirinezhad, Mahshad Sabri, Mehdi Mehrani, Homa Taheri, Robert Siegel, Neeraj Shah, Michael Nanna, Diaa Hakim, Kaveh Hosseini

**Affiliations:** ^1^ Tehran Heart Center, Cardiovascular Disease Research Institute Tehran University of Medical Sciences Tehran Iran; ^2^ School of Medicine Tehran University of Medical Sciences Tehran Iran; ^3^ Smidt Heart Institute Cedars‐Sinai Medical Center Los Angeles California USA; ^4^ Independence Health estmoreland Hospital Greensburg Pennsylvania USA; ^5^ Section of Cardiovascular Medicine Yale University School of Medicine New Haven CT USA; ^6^ Brigham and Women's Hospital Harvard Medical School Boston Massachusetts USA

**Keywords:** aortic stenosis, coronary artery disease, meta‐analysis, percutaneous coronary intervention, systematic review, TAVI, transcatheter aortic valve implantation

## Abstract

**Background:**

Transcatheter aortic valve implantation (TAVI) has been increasingly used in patients with severe aortic stenosis (AS). Since coronary artery disease (CAD) is common among these patients, it is crucial to choose the best method and timing of revascularization. This study aims to compare different timing strategies of percutaneous coronary intervention (PCI) in patients with severe AS undergoing TAVI to clarify whether PCI timing affects the patients' outcomes or not.

**Methods:**

A frequentist network meta‐analysis was conducted comparing three different revascularization strategies in patients with CAD undergoing TAVI. The 30‐day all‐cause mortality, in‐hospital mortality, all‐cause mortality at 1 year, 30‐day rates of myocardial infarction (MI), stroke, and major bleeding, and the need for pacemaker implantation at 6 months were analyzed in this study.

**Results:**

Our meta‐analysis revealed that PCI during TAVI had higher 30‐day mortality (RR = 2.46, 95% CI = 1.40–4.32) and in‐hospital mortality (RR = 1.70, 95% CI = [1.08–2.69]) compared to no PCI. Post‐TAVI PCI was associated with higher 1‐year mortality compared to other strategies. While no significant differences in major bleeding or stroke were observed, PCI during TAVI versus no PCI (RR = 3.63, 95% CI = 1.27–10.43) showed a higher rate of 30‐day MI.

**Conclusion:**

Our findings suggest that among patients with severe AS and CAD undergoing TAVI, PCI concomitantly with TAVI seems to be associated with worse 30‐day outcomes compared with no PCI. PCI after TAVI demonstrated an increased risk of 1‐year mortality compared to alternative strategies. Choosing a timing strategy should be individualized based on patient characteristics and procedural considerations.

## Graphical abstract

In patients with severe aortic stenosis (AS) and coronary artery disease (CAD) undergoing transcatheter aortic valve implantation (TAVI), the optimal timing of percutaneous coronary intervention (PCI) remains contentious. We conducted a network meta‐analysis comparing different PCI timing strategies (pre‐TAVI PCI, PCI during TAVI, and post‐TAVI PCI). Findings indicate PCI during TAVI is associated with higher in‐hospital and 30‐day mortality compared to no PCI. Conversely, PCI after TAVI correlates with elevated 1‐year all‐cause mortality. Individualized decision‐making, considering patient characteristics and procedural nuances, is crucial.

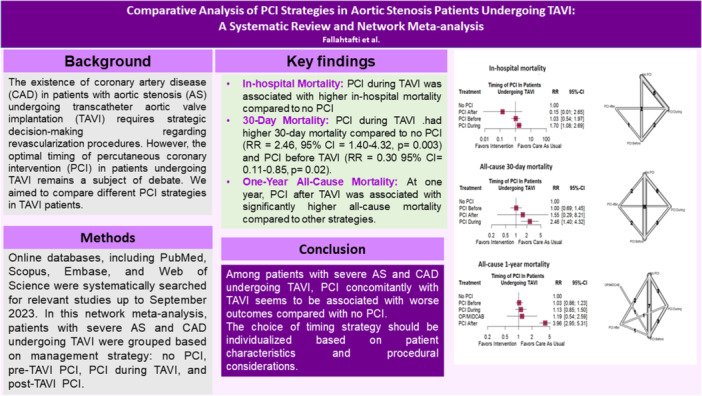

## 1 Introduction

Aortic stenosis (AS) is the most common valvular disease in developed countries, burdening nine million people globally [1], and its prevalence increases as populations age: from 20% in patients between 65% and 75% to 48% in patients over 85 years of age [2, 3]. Although surgical valve replacement is considered to be the standard treatment of severe AS, this approach could not be applied to all patients, especially those with multiple comorbidities and higher surgical risks [4].

Transcatheter aortic valve implementation (TAVI) has increasingly been used since its emergence and has grown to be an acceptable alternative to surgical valve replacement in AS patients with noninferior results [5, 6, 7] and has been used with success not only in high‐risk patients but also in intermediate and low‐risk groups [5, 6, 7, 8, 9]. It is estimated that so far, over 250 000 successful procedures have been performed, and the number is projected to surpass 280 000 implants in 2025 alone [10].

Coronary artery disease (CAD) and AS commonly coexist [11]; it is estimated that 40‐75% of patients undergoing TAVI have CAD [5, 8, 12]. It is suggested that this association is mostly due to the similar risk factors of both entities, including age, male sex, hyperlipidemia, and underlying inflammatory mechanisms [3]. Since AS can induce myocardial ischemia independent of pre‐existing CAD [13] and CAD could lead to suboptimal outcomes after TAVI [14], the management of CAD in patients undergoing TAVI poses challenges that need to be considered, including whether CAD is severe enough to ensure the need for revascularization, and the optimal timing for revascularization. To date, there is no consensus regarding the management of CAD in patients undergoing TAVI [15]. Percutaneous coronary intervention (PCI), as a nonsurgical revascularization method addressing CAD, can be performed either before TAVI, after TAVI, or simultaneously as a combined procedure. While each strategy has its own potential risks and advantages, it is crucial to determine the optimal timing strategy.

This systematic review and network meta‐analysis aims to compare different PCI timing strategies in patients with severe AS combined with CAD undergoing TAVI.

## 2 Materials and Methods

This systematic review and network meta‐analysis adhered to the Preferred Reporting Items for Systematic Reviews and Meta‐Analyses (PRISMA) criteria [16]. The protocol used for this analysis was submitted to the PROSPERO online database with the registration ID PROSPERO CRD42023477451 (https://www.crd.york.ac.uk/prospero/display_record.php?ID=CRD42023477451).

### 2.1 Search Strategy, Selection Criteria, and Data Extraction

A comprehensive systematic search strategy was created and conducted in PubMed, Scopus, Web of Science, and EMBASE in September 2023, from their inception to the search date (Supporting Information S1). Reference lists of eligible studies and reviews on the topic were also screened for potential additions.

All studies, whether clinical trials or observational studies, that compared different methods and timings of revascularization in patients with confirmed CAD who had undergone TAVI procedure, regardless of the timing of their coronary revascularization procedure, were included in the analysis. Results from noncomparative studies were not considered for the network meta‐analysis model, but they were utilized to estimate proportion (see Section 2.4). Studies that involved aortic valve reconstruction/replacement using methods other than TAVI, conference abstracts, reviews, non‐English language, and animal studies were excluded.

The search strategy was designed and implemented using the Rayaan platform, an advanced tool for systematic review management [17]. Two researchers (P.F. and A.G.) separately examined the titles and abstracts of the obtained citations to determine which studies were eligible. The full texts of the selected citations were then individually assessed by the same two investigators. Any disagreements that arose were resolved through mutual agreement at each stage. The data from the citations that remained on the final list after the full‐text review was collected by the same two authors, who also cross‐verified each other's work to ensure consistency.

In the next step, data from the included studies were extracted by three authors (E.K., M.S., A.S.) using a standardized data collection sheet in Microsoft Excel (Microsoft Corporation, Redmond, WA, USA). The extracted data encompassed various variables, including author names, publication years, study designs, sample sizes, follow‐up duration, participant ages, gender distribution, baseline coronary risk scores of each study arm (including SYNTAX and Logistic EuroSCORE), type of transcatheter heart valve, timing of revascularization procedure, and data regarding primary and secondary outcomes.

### 2.2 Outcomes

Our primary study outcome was 30‐day all‐cause mortality, as it was reported in more studies and had the least amount of missing data. For secondary outcomes, we gathered, analyzed, and reported in‐hospital mortality, 30‐day rates of myocardial infarction (MI), stroke, and major bleeding, the need for pacemaker implantation at 6 months, and all‐cause mortality at 1 year post‐TAVI procedure.

### 2.3 Risk of Bias Assessment

The risk of bias was evaluated independently by three authors (E.K., M.S., A.S.) using the critical appraisal tool for prognosis and randomized controlled trials (RCT) developed by the Centre for Evidence‐Based Medicine (CEBM) (Available from https://www.cebm.ox.ac.uk/resources/ebm-tools/critical-appraisal-tools). Three authors (E.K., M.S., A.S.) assigned evaluated studies in each category, and disagreements were resolved by consensus between the authors.

### 2.4 Statistical Analysis

Network meta‐analysis models were constructed using a frequentist approach. In short, this approach utilizes graph theoretical models previously developed for electrical networks [18, 19] and allows for indirect comparisons between various treatment strategies, making it possible to compare the effects of several interventions simultaneously [20]. The concept of frequentism is a theoretical approach used to understand the likelihood of an event E. In frequentist interpretations, the probability of E is determined by the frequency at which E is anticipated to happen when a process is repeated numerous times [21].

All reported outcomes in our study were of a binary nature, so to pool treatment effect estimates, relative risk (RR) for each outcome was calculated and pooled using the Mantel–Haenszel method. Statistical heterogeneity was assessed using the *I* ^2^ statistic and the between‐study variance (*τ* ^2^) in random‐effects models. The Sidik–Jonkman estimator was employed to determine the heterogeneity variance *τ* ^2^ [22, 23]. As it was predicted that significant heterogeneity would be present between studies, a random effects model was utilized to estimate and compare treatment effect sizes. To evaluate network consistency and assess direct/indirect evidence agreement, node‐splitting was used [24]. We used the Egger test to formally investigate reporting bias. This test examines the symmetry of funnel plots. If the test results were not statistically significant, it was determined that the risk of reporting bias was minimal [25]. For studies without comparative arms, the proportion of outcomes reported was pooled using a generalized linear mixed‐effects model with logit transformations. All statistical analyses were conducted using the R software [26] (R for Windows, version 4.1.3, Vienna, Austria) and R Studio version 1.1.463 (Posit PBC, Boston, MA, USA), utilizing packages tidyverse [27], meta [28], netmeta [29], and robvis [30].

## 3 Results

### 3.1 Study Selection and Baseline Characteristics

The initial search yielded a total of 5430 articles. After removing duplicates, unique records remained. The screening process involved an assessment of titles and abstracts for relevance, resulting in the exclusion of 3035 records that did not meet the predefined criteria.

**Figure 1:**
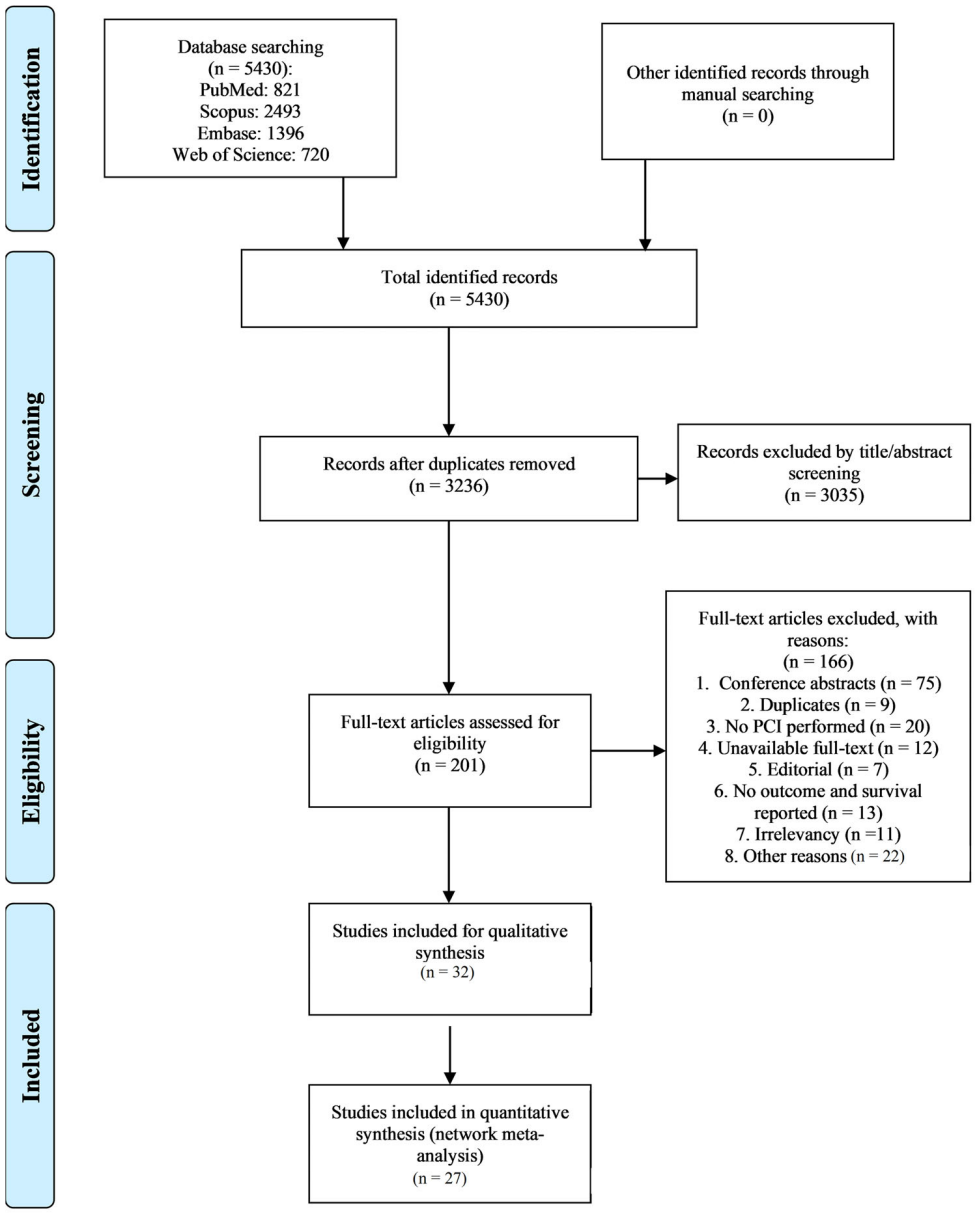
PRISMA flow diagram.

**Figure 2:**
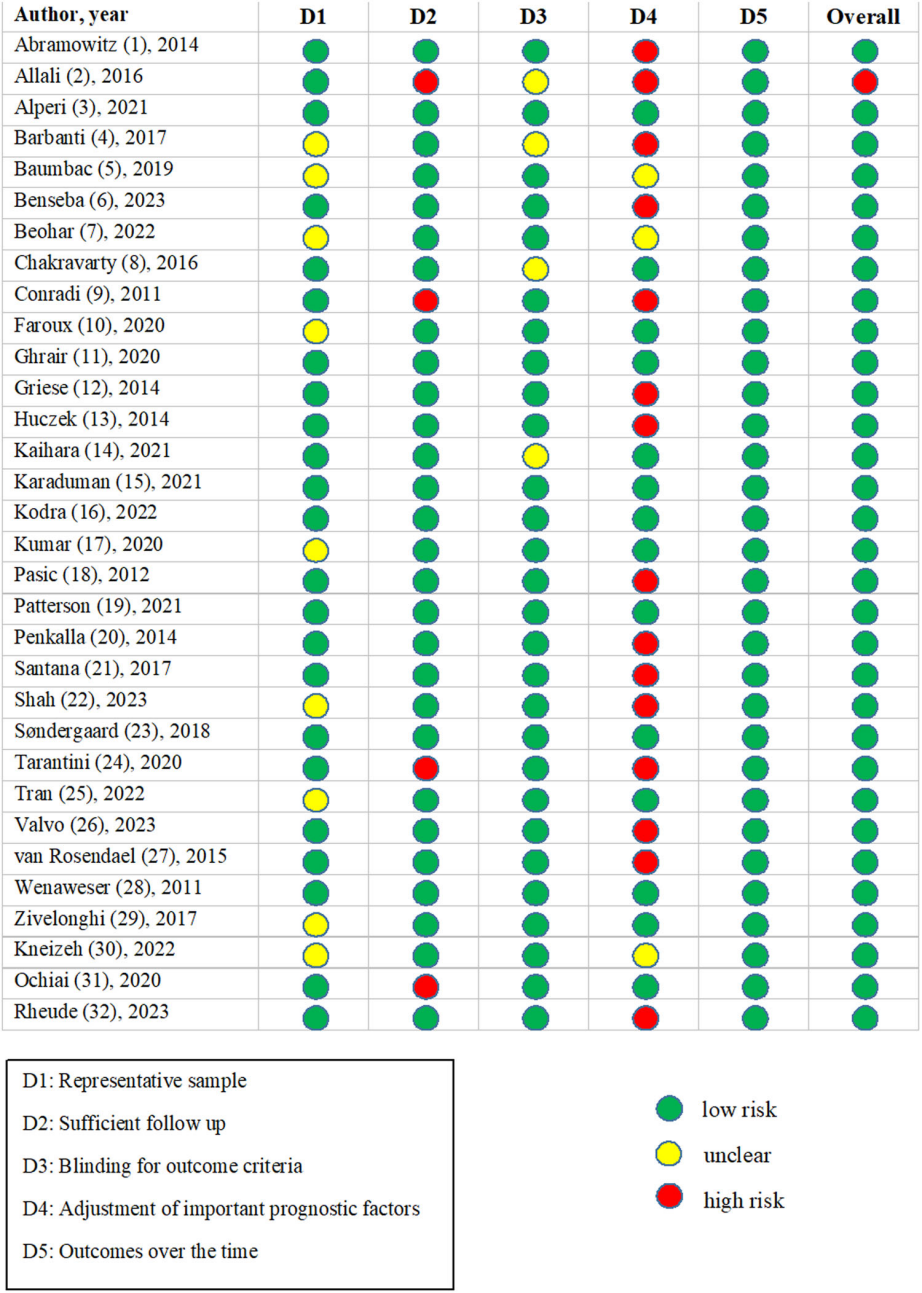
Risk of bias, quality assessment was evaluated using the critical appraisal tool for prognosis and randomized controlled trials (RCT) developed by the Centre for Evidence Based Medicine (CEBM).

The remaining 201 articles underwent a full‐text assessment to determine eligibility. A total of 32 articles met the eligibility criteria for inclusion in the systematic review. Twenty‐seven studies were included in the final network meta‐analysis. The study selection process is visually represented in the PRISMA flow diagram (Figure 1). Excluded studies at the full‐text stage were documented along with the reasons for their exclusion (Supporting Information S1: Table S1). A list of included studies is provided in Table 1 and Supporting Information S1: Table S2, along with key characteristics, such as author names, publication year, and study design. The included studies were published between 2010 and 2023 with variations in sample size and study duration. The selected studies encompassed a variety of study designs, including clinical trials and observational studies. The details of the quality assessment of included studies are presented in Figure 2.

**Table 1:** Baseline characteristics of the included studies (Part 1).

First author, year	Study type	Test group treatment	Control treatment	Test Group (n)	Control size (*n*)
Abramowitz [31], 2014	Retrospective cohort	PCI before or during TAVI	No PCI	61	83
Allali [32], 2016	Retrospective observational	PCI after TAVI	—	17	—
Alperi [33], 2021	Prospective cohort	PCI before or during TAVI	—	156	—
Barbanti [34], 2017	Prospective cohort	PCI during TAVI	No PCI	51	83
Baumbac [35], 2019	Prospective cohort	PCI during TAVI	—	112	—
Benseba [36], 2023	Retrospective cohort	PCI before TAVI	No PCI	425	598
Beohar [37], 2022	Retrospective observational	PCI before TAVI	—	18	—
Chakravarty [38], 2016	Case‐control	PCI before, during, or after TAVI	No PCI	128 (post‐match)	128 (post‐match)
Conradi [39], 2011	Clinical trial	PCI before or during TAVI	—	28	—
Faroux [40], 2020	Retrospective observational	PCI before or during TAVI	—	1197	—
Ghrair [41], 2020	Retrospective cohort	PCI during TAVI	No PCI	812	812
Griese [42], 2014	Prospective cohort	PCI before or during TAVI	No PCI	65	346
Huczek [43], 2014	Retrospective cohort	PCI before TAVI	No PCI	169	293
Kaihara [44], 2021	Retrospective observational	PCI before TAVI	—	29	—
Karaduman [45], 2021	Retrospective cohort	PCI before, during, or after TAVI	No PCI	65	52
Kneizeh [46], 2022	Retrospective cohort	PCI before or during TAVI		21	
Kodra [47], 2022	Retrospective observational	PCI before or during TAVI	—	276	—
Kumar [48], 2020	Prospective cohort	PCI during or after TAVI	PCI before TAVI	53	327
Ochiai [49], 2020	Retrospective cohort	PCI before or after TAVI	PCI during TAVI	181	77
Pasic [50], 2012	Prospective observational	PCI during TAVI	—	46	—
Patterson [12], 2021	RCT	PCI before TAVI	No PCI	119	116
Penkalla [51], 2014	Prospective cohort	concomitant PCI	No PCI	76	232
Rheude [52], 2023	Retrospective cohort	PCI before or after TAVI	PCI during TAVI	1209	394
Santana [53], 2017	Retrospective observational	PCI before or TAVI	—	123	—
Shah [54], 2023	Retrospective cohort	PCI before TAVI	No PCI	144	138
Søndergaard [55], 2018	Retrospective cohort	PCI before TAVI	No PCI	123	42
Tarantini [56], 2020	Retrospective cohort	PCI after TAVI	No PCI	68	1868
Tran [57], 2022	Retrospective cohort	PCI before TAVI	PCI during TAVI	5000	843
Valvo [58], 2023	Retrospective cohort	PCI during TAVI	No PCI	151	151
van Rosendael [59], 2015	Retrospective observational	PCI before TAVI	—	96	—
Wenaweser [60], 2011	Retrospective cohort	PCI before or during TAVI	No PCI	59	197
Zivelonghi [61], 2017	Prospective cohort	PCI before or during TAVI	No PCI	34	89

Abbreviations: *n*, number; PCI, percutaneous coronary intervention; RCT, randomized controlled trial; TAVI, trans‐catheter aortic valve implantation.

### 3.2 30‐Day All‐Cause Mortality

**Figure 3:**
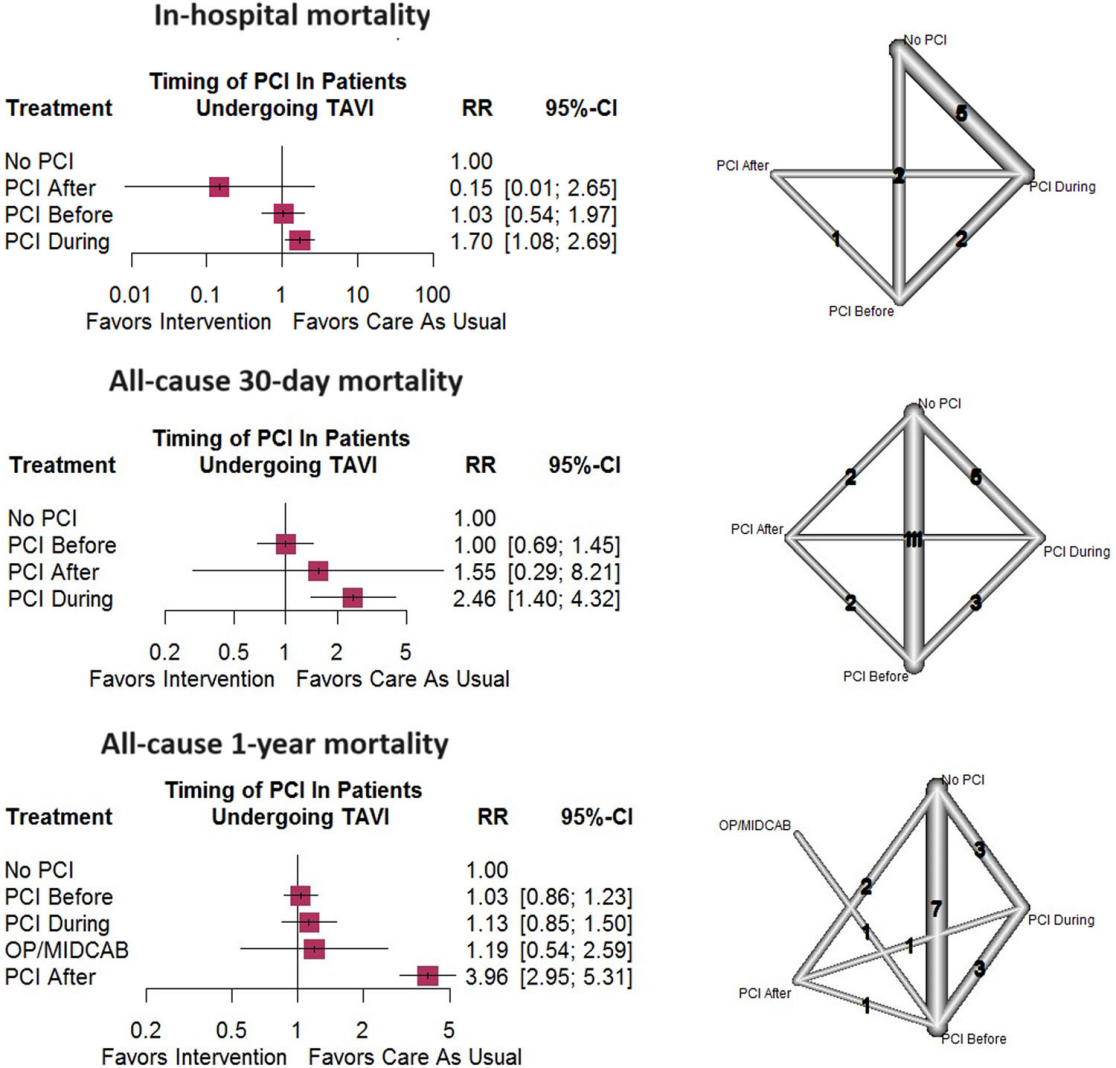
Network meta‐analysis of 30‐day all‐cause mortality, in‐hospital mortality, and one‐year mortality after TAVI.

A 30‐day mortality following TAVI was reported in seven of the included noncomparative studies. Pooled analysis revealed that the mortality rate 30 days following the procedure was 2.0% (95% CI = [1.0–7.0]) (Figure S1). Regarding the association between the timing of PCI and 30‐day mortality risk, these direct comparisons have been analyzed in some of the eligible investigations: (1) PCI before versus during TAVI (three studies), (2) PCI before versus after TAVI (two studies), (3) PCI after versus during TAVI (one study), (4) PCI before TAVI versus no PCI (11 studies), (5) PCI after TAVI versus no PCI (two studies), and (6) PCI during TAVI versus no PCI (five studies). Direct comparisons indicated that PCI before TAVI had a lower 30‐day mortality risk compared to PCI during TAVI (RR = 0.30, 95% CI = [0.11–0.85]) in opposition to post‐replacement coronary intervention (RR = 1.67, 95% CI = [0.28–10.15]) (Figure S2). Moreover, PCI during TAVI seemed to increase 30‐day mortality risk compared to no PCI group (RR = 2.42, 95% CI = [1.35–4.35]). Network meta‐analysis confirmed the mentioned findings. As shown in Figure 3, network estimates were not in favor of performing PCI during TAVI when compared to the no PCI group (RR = 2.46, 95% CI = [1.40–4.32]). However, PCI before (RR = 1.00, 95% CI = [0.69–1.45]) and after TAVI (RR = 1.55, 95% CI = [0.29–8.21]) did not show a significant association with an enhanced risk of postprocedure 30‐day mortality (Figure 3). Network inconsistency (*p* = 0.7896) and publication bias (*p* = 0.4083) were not significant based on proper statistical tests.

### 3.3 In‐Hospital Mortality

In general, five noncomparative and ten comparative studies reported in‐hospital mortality. Comparisons analyzed in the included studies consisted of (1) no PCI versus PCI before TAVI (two studies), (2) no PCI versus PCI during TAVI (five studies), (3) PCI before versus PCI during TAVI (two studies), and (4) PCI before versus PCI after (one study). Direct comparison between PCI before versus during TAVI (RR = 0.59, 95% CI = [0.23–1.50]) and PCI before TAVI versus no PCI (RR = 1.02, 95% CI = [0.46–2.26]) demonstrated no noteworthy differences regarding in‐hospital mortality (Figure S3). However, PCI during TAVI was associated with higher in‐hospital mortality compared to the no PCI group (RR = 1.70, 95% CI = 1.04–2.77). Network meta‐analysis indicated no significant difference while performing PCI before compared to no PCI (RR = 1.03, 95%CI = [0.54–1.97]), and also PCI before versus during TAVI (RR = 0.61, 95% CI = [0.31–1.19]). Similar to direct comparison, in the network meta‐analysis, PCI during TAVI was associated with higher in‐hospital mortality (RR = 1.70, 95% CI = [1.08–2.69]) compared to no PCI (Figure 3). Network inconsistency (*p* = 0.6066) was not significantly observed between direct and indirect estimates.

### 3.4 1‐Year All‐Cause Mortality

A 1‐year mortality following TAVI was reported in four of the included noncomparative studies. Pooled analysis showed that the mortality rate a year after the procedure was around 8.0% (95% CI = [1.0–40.0]) (Figure S1). Regarding 1‐year mortality, patients were divided into two groups directly comparing: (1) PCI before versus during TAVI (three studies), (2) PCI before versus after TAVI (one study), (3) PCI after versus during TAVI (one study), (4) PCI before TAVI versus no PCI (seven studies), (5) PCI after TAVI versus no PCI (two studies), (6) PCI during TAVI versus no PCI (three studies), and (7) OP/MIDCAB (off pump−minimally invasive direct coronary artery bypass grafting) versus PCI before TAVI (one study). Meta‐analysis of the direct comparisons revealed that undergoing PCI after TAVI led to a higher risk of mortality at 1‐year follow‐up compared to the control no PCI group (RR = 3.96, 95% CI = [2.95–5.31]) (Figure S4). Other analyses did not result in a significant finding. Network meta‐analysis, pooling direct and indirect estimates, demonstrated a meaningful increase in the risk of 1‐year mortality in those admitted to undergo PCI following TAVI compared to no PCI (RR = 3.96, 95% CI = [2.95–5.31]), PCI before (RR = 3.84, 95% CI = [2.73–5.41]), and PCI during valvular replacement (RR = 3.51, 95% CI = [2.33–5.29]). Pre‐TAVI PCI and concomitant PCI were not significantly dissimilar in terms of 1‐year mortality (RR = 0.91, 95% CI = [0.66–1.26]) (Figure 3). Network inconsistency (*p* = 0.1743) and publication bias (*p* = 0.4584) were not significant based on the statistical analysis.

### 3.5 Major Bleeding

**Figure 4:**
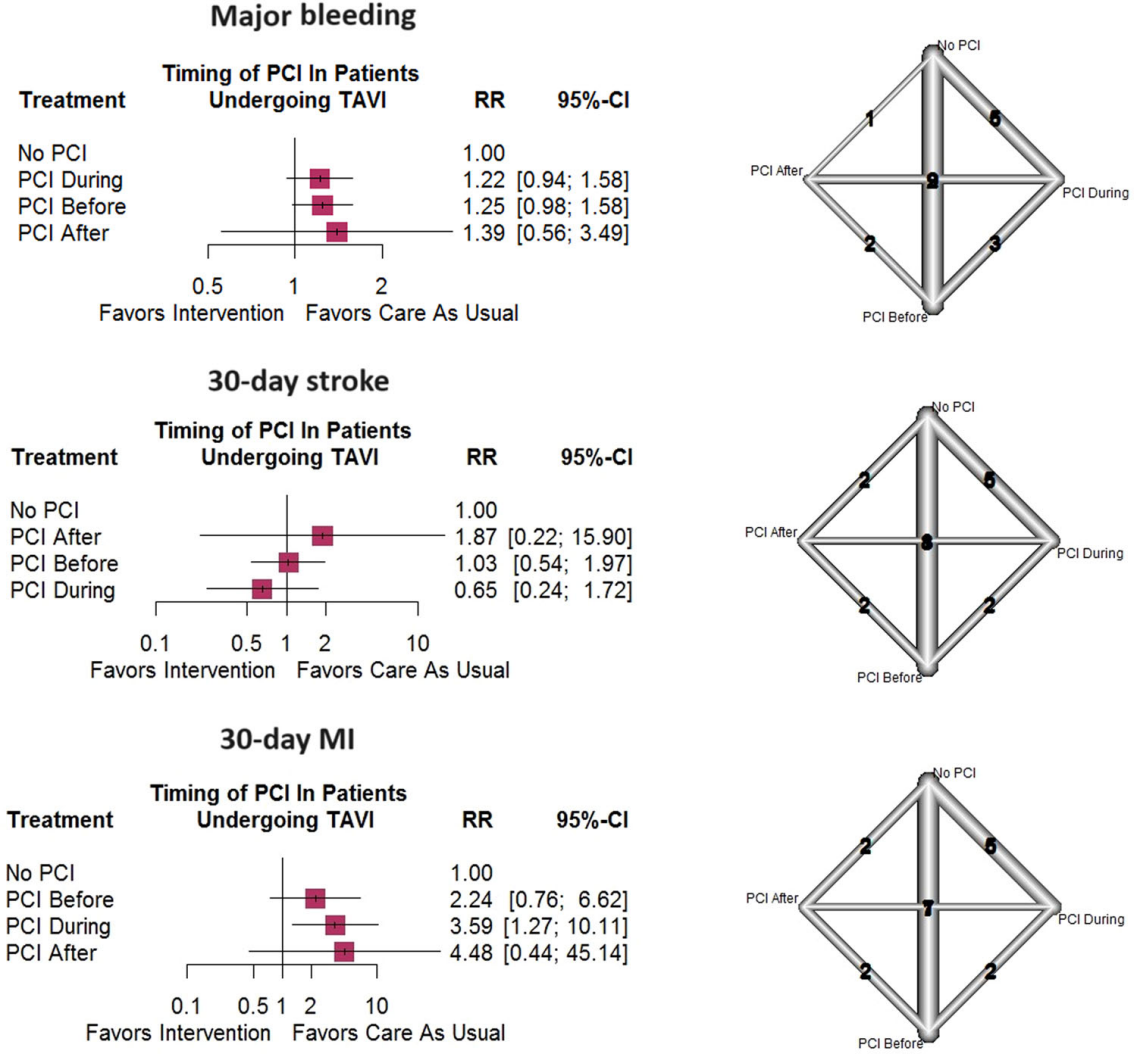
Network meta‐analysis of major bleeding, 30‐day stroke, and 30‐day MI after TAVI.

Direct comparisons between (1) PCI before versus during TAVI (three studies), (2) PCI before versus after TAVI (two studies), (3) PCI after versus during TAVI (two studies), (4) PCI before TAVI versus no PCI (nine studies), (5) PCI after TAVI versus no PCI (one study), and (6) PCI during TAVI versus no PCI (five studies) were analyzed in the included studies. Meta‐analyses of the direct comparisons indicated no noteworthy differences between various PCI timings and control populations (Figure S5). Moreover, PCI before (RR = 1.25, 95% CI =[0.98–1.58]), during (RR = 1.22, 95% CI = [0.94–1.58]), or after (RR = 1.39, 95% CI = [0.56–3.49]) TAVI were not observed to induce postoperative bleeding compared to the no PCI group (Figure 4). A noteworthy inconsistency was observed between direct and indirect estimates (*p* = 0.0320); however, no publication bias was detected (*p* = 0.0605).

### 3.6 Stroke and Myocardial Infarction

The two most important components of major adverse cardiovascular events (MACE) were postoperative stroke and MI. About stroke, direct comparisons included: (1) PCI before versus during TAVI (two studies), (2) PCI before versus after TAVI (two studies), (3) PCI after versus during TAVI (one study), (4) PCI before TAVI versus no PCI (eight studies), (5) PCI after TAVI versus no PCI (two studies), and (6) PCI during TAVI versus no PCI (five studies). Postoperative MI was compared between (1) PCI before versus during TAVI (two studies), (2) PCI before versus after TAVI (two studies), (3) PCI after versus during TAVI (one study), (4) PCI before TAVI versus no PCI (seven studies), (5) PCI after TAVI versus no PCI (two studies), and (6) PCI during TAVI versus no PCI (five studies) in the eligible articles. Both traditional and network meta‐analyses indicated no significant association between PCI timing and post‐TAVI stroke (Figures 4 and S6). However, PCI during TAVI seemed to have a meaningful impact on the postprocedure MI incidence compared to no PCI (RR = 3.63, 95% CI = [1.27–10.43]) but not PCI before (RR = 0.50, 95% CI = [0.03–7.77]) or after TAVI (RR = 2.36, 95% CI = [0.05–111.74]), based on direct comparisons (Figure S7). Network estimates confirmed this association, too (PCI during vs. no PCI: RR = 3.59, 95% CI = [1.27–10.11]) (Figure 4). Both stroke (*p* = 0.8130) and MI incidence (*p* = 0.8394) meta‐analyses did not show a meaningful inconsistency between direct and indirect methods. Moreover, publication bias was not significant either.

### 3.7 Permanent Pacemaker Placement

Another secondary endpoint analyzed was the post‐TAVI 6‐month incidence of permanent pacemaker placement. Comparisons included in this regard were: (1) PCI before versus during TAVI (one study), (2) PCI before versus after TAVI (one study), (3) PCI after versus during TAVI (one study), (4) PCI before TAVI versus no PCI (nine studies), (5) PCI after TAVI versus no PCI (one study), and (6) PCI during TAVI versus no PCI (six studies). A traditional meta‐analysis demonstrated no significant differences between PCI timings and no PCI (Figure S8). On the other hand, network meta‐analysis revealed that PCI before TAVI had a slightly increased risk of pacemaker placement following the operation compared to PCI during TAVI. On the contrary, all timings were not meaningfully different with the no PCI control group (Figure 4). Network inconsistency (*p* = 0.6213) and publication bias (*p* = 0.3943) were not significant based on the statistical analysis.

### 3.8 Publication Bias

Egger's regression test and Begg's funnel plot were utilized to evaluate publication bias in all network meta‐analyses. All the performed analyses did not indicate any publication bias (Figure S9).

## 4 Discussion

With the increasing prevalence of TAVI and a growing number of patients opting for this procedure, the management of CAD in this population has become a crucial and pressing question. The rising trend of younger patients with AS choosing TAVI over traditional surgical interventions, driven by lower surgical risks and extended life expectancy, adds complexity to addressing CAD [62]. However, determining the best time for performing PCI in patients undergoing TAVI is a complex dilemma. In this study, we aimed to compare clinical outcomes of different timing strategies. Our systematic review and network meta‐analysis show that concomitant PCI was associated with higher in‐hospital mortality and 30‐day mortality compared to no PCI group, while PCI following TAVI was associated with higher 1‐year mortality compared to other strategies. A higher rate of 30‐day MI was observed in PCI during TAVI compared to no PCI. A 30‐day life‐threatening bleeding, 30‐day stroke, and the need for pacemaker implantation after 6 months showed no significant differences between the three strategies.

While, in our study, PCI before TAVI was associated with lower 30‐day mortality compared to concomitant PCI, in the study of Wenawesar et al., the mortality rate at 30 days was 8.7%, and 11.1% in the staged and concomitant PCI and TAVI groups, respectively, which implied no statistically significant difference [60]. The study findings demonstrated a higher incidence of substantial access‐related complications and life‐threatening bleeding in the staged PCI and TAVI group as compared to the concomitant PCI and TAVI group; the difference was not statistically significant [60].

Although the majority of PCIs are conducted before TAVI due to the concerns surrounding the possibility of ischemic complications during the valve implantation procedure, the study of Lunardi et al. has shown that both overall survival and MACE‐free survival at 24 months were notably lower in patients who underwent PCI before TAVI compared with patients undergoing PCI after TAVI [63]. Also, a meta‐analysis revealed that revascularization strategies involving PCI before TAVI do not yield improved short‐ or mid‐term mortality outcomes [64]. Additionally, in a study by Altibi et al., pre‐TAVI PCI showed no improved 30‐day or 1‐year mortality rates, and it was associated with an elevated risk of life‐threatening bleeding 30 days post‐TAVI [64].

Since all pathologies are addressed simultaneously in concomitant PCI and TAVI, it could theoretically reduce postoperative MI, use one transarterial access for both procedures at the same time, and hence decrease the occurrence of bleeding and vascular complications [15, 51, 65]. However, according to some studies, these potential benefits may not be practical in the real world. Rates of peri‐procedural mortality, vascular complications, and MI were reported to be increased in concomitant TAVI and PCI [60, 66]. The results of studies comparing staged and concomitant PCI with TAVI are conflicting. Pasic et al. have shown improved 1‐ and 2‐year survival rates and a favorable 30‐day mortality rate (4.3%), suggesting promising results of simultaneous TAVI and PCI [50]. In addition, a retrospective cohort study indicated a 30‐day mortality rate of 7.1%, as well as no occurrences of periprocedural MI or stroke among patients treated with a single‐stage procedure [39]. Although, in our analysis, in‐hospital mortality and MI were not different between staged and concomitant PCI, these outcomes were worse in concomitant PCI compared to no PCI control. Concomitant PCI showed a higher risk of 30‐day mortality compared to PCI before TAVI and no PCI. In contrast, a prior cohort study found no statistical difference between patients who underwent concomitant PCI with TAVI compared to those who got TAVI procedure alone concerning 30‐day outcomes, although the rate of 30‐day MI was higher in the hybrid group (12 vs. 4%) [42]. It has been shown elsewhere that 30‐day outcomes were worsened when PCI was performed during the same hospital admission [67]. Although, to date, several reports have demonstrated that PCI after valve transplantation is usually feasible and safe [68, 69], data about the outcome of performing PCI following TAVI is limited. As mentioned previously, a retrospective analysis of short‐ and long‐term outcomes of patients undergoing PCI before or after TAVI revealed that post‐TAVI PCI was not accompanied by higher risks of peri‐procedural complications and also had more favorable overall survival and MACE‐free survival compared with pre‐TAVI PCI [63]. TAVI alleviates left ventricular pressure overload, resulting in improved systemic perfusion. The observed lower risk of peri‐procedural hazards, such as stroke rates in patients receiving PCI after TAVI, may be attributed to the immediate beneficial hemodynamic impact of TAVI [70, 71, 72]. Another study by Rheude et al. demonstrated that conducting PCI after TAVI is associated with improved 2‐year clinical outcomes [52]. Our results showed a higher rate of 1‐year mortality in PCI after TAVI compared to the other two strategies. Considering all these findings, the technical challenges of post‐TAVI PCI should be in mind. Prior studies have shown that it is possible, albeit unlikely, that the valve struts compromise coronary artery cannulation by being dislodged during catheter manipulation [32].

## 5 Conclusion

In conclusion, our findings suggest that different PCI timing strategies are not significantly associated with in‐hospital mortality and 30‐day mortality. However, PCI concomitantly with TAVI seems to be associated with worse in‐hospital mortality, 30‐day mortality, and MI compared with no PCI group. The performance of PCI after TAVI demonstrated an increased risk of 1‐year mortality compared to all alternative strategies. While our findings align with some previous studies, discrepancies exist, emphasizing the complexity of this decision. The choice between pre‐TAVI, simultaneous, or post‐TAVI PCI should be tailored to individual patient characteristics, considering the potential benefits and challenges associated with each strategy.

## 6 Strengths and Limitations

Our systematic review and network meta‐analysis provide a comprehensive examination of the optimal timing of PCI in TAVI, addressing both long‐term and in‐hospital outcomes. This thorough investigation enhances our understanding of the subject and offers valuable insights for clinical decision‐making. However, our study faces certain limitations that warrant consideration. The first is the scarcity of clinical trials focusing on the optimal timing of PCI in TAVI, highlighting a gap in the existing evidence. Additionally, the limited number of studies directly comparing the three different timings of PCI hinders the depth of our comparative analysis. The paucity of post‐TAVI PCI studies further restricts the scope of our findings. The inclusion of studies involving first‐generation TAVI devices in our meta‐analysis may introduce potential bias due to advancements in newer‐generation transcatheter heart valves. Given that the literature predominantly encompasses various generations of valve devices and lacks studies exclusively focusing on newer‐generation subgroups, we recognize this as a limitation in our study. Moreover, a limited number of studies reported hazard ratios (HR), affecting our ability to comprehensively analyze the risk associations related to different PCI timings in TAVI.

## Supporting information

FIGURE S1 Rates of 30‐day all‐cause mortality, in‐hospital mortality, 1‐year mortality, and 30‐day MI.

FIGURE S2 Subgroup analysis of 30‐day all‐cause mortality.

FIGURE S3 Subgroup analysis of in‐hospital mortality.

FIGURE S4 Subgroup analysis of 1‐year all‐cause mortality.

FIGURE S5 Subgroup analysis of major bleeding.

FIGURE S6 Subgroup analysis of 30‐day stroke.

FIGURE S7 Subgroup analysis of 30‐day myocardial infarction.

FIGURE S8 Subgroup analysis of 6 months permanent pacemaker placement.

FIGURE S9 Funnel plots representing publication bias.

Supporting information.

## Data Availability

The data that support the findings of this study are available on request from the corresponding author. The data are not publicly available due to privacy or ethical restrictions.
