# Supplementary figures and images for: Comparative Analysis of PCI Strategies in Aortic Stenosis Patients Undergoing TAVI: A Systematic Review and Network Meta‐Analysis
Source: Clin Cardiol. 2024 Jul 26;47(8):e24324. doi: 10.1002/clc.24324 (PMC11272956; doi:10.1002/clc.24324)

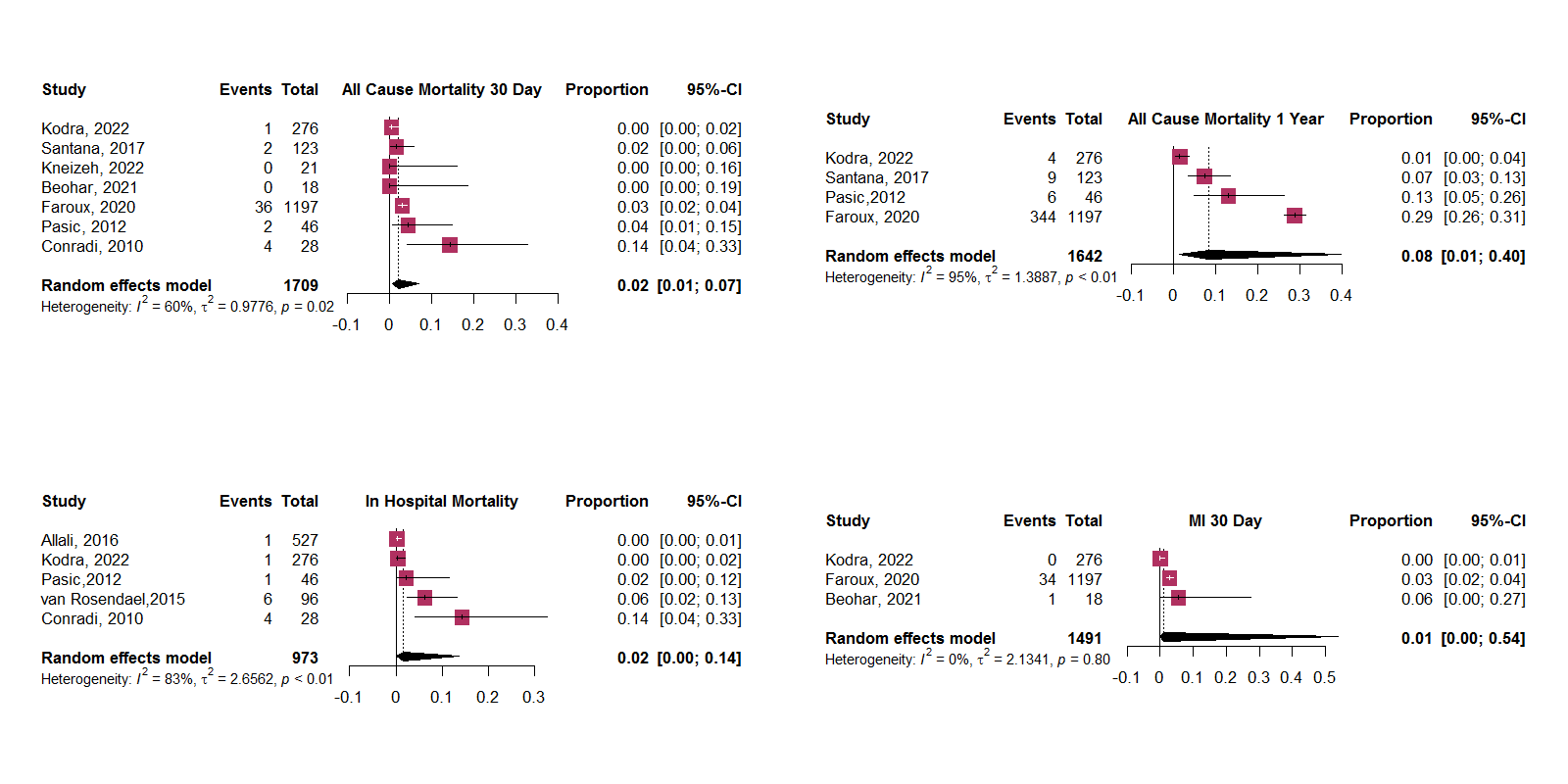

Supplement: Supplementary file 1 — FIGURE S1 Rates of 30‐day all‐cause mortality, in‐hospital mortality, 1‐year mortality, and 30‐day MI. [file CLC-47-e24324-s005.png]

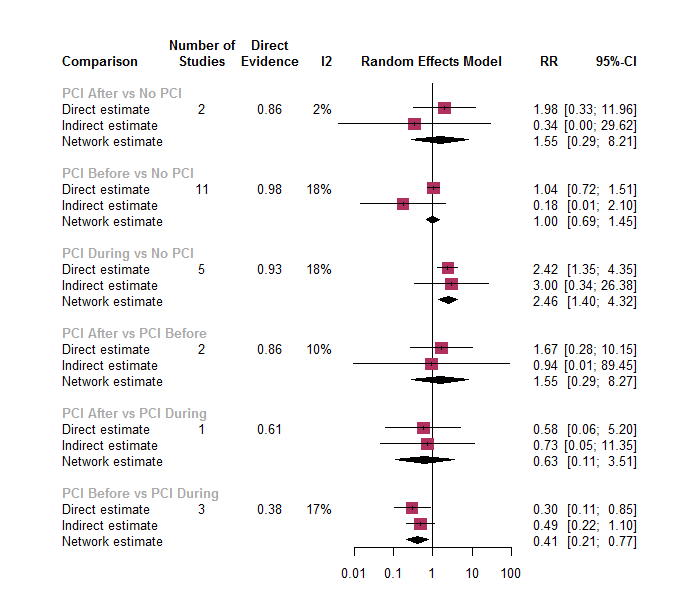

Supplement: Supplementary file 2 — FIGURE S2 Subgroup analysis of 30‐day all‐cause mortality. [file CLC-47-e24324-s010.png]

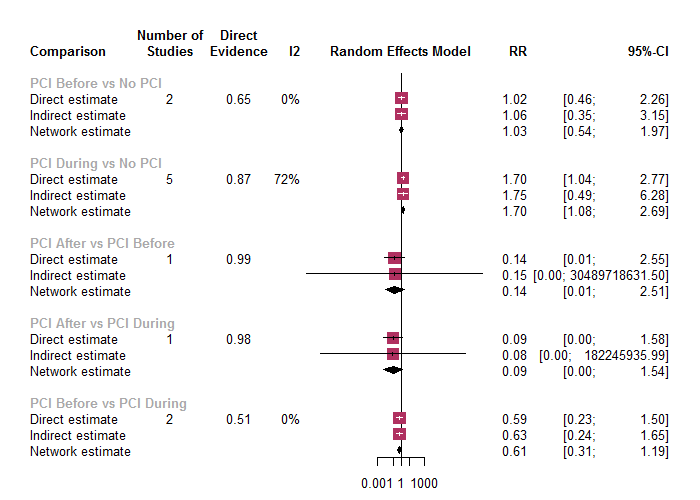

Supplement: Supplementary file 3 — FIGURE S3 Subgroup analysis of in‐hospital mortality. [file CLC-47-e24324-s008.png]

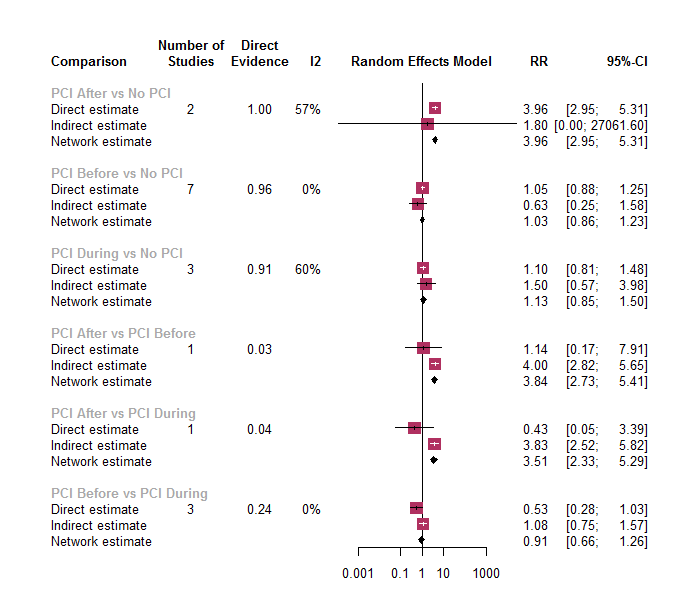

Supplement: Supplementary file 4 — FIGURE S4 Subgroup analysis of 1‐year all‐cause mortality. [file CLC-47-e24324-s009.png]

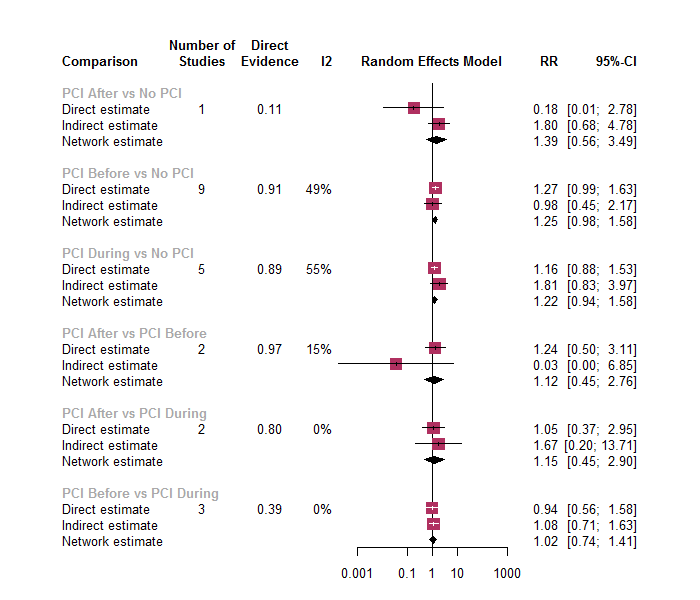

Supplement: Supplementary file 5 — FIGURE S5 Subgroup analysis of major bleeding. [file CLC-47-e24324-s001.png]

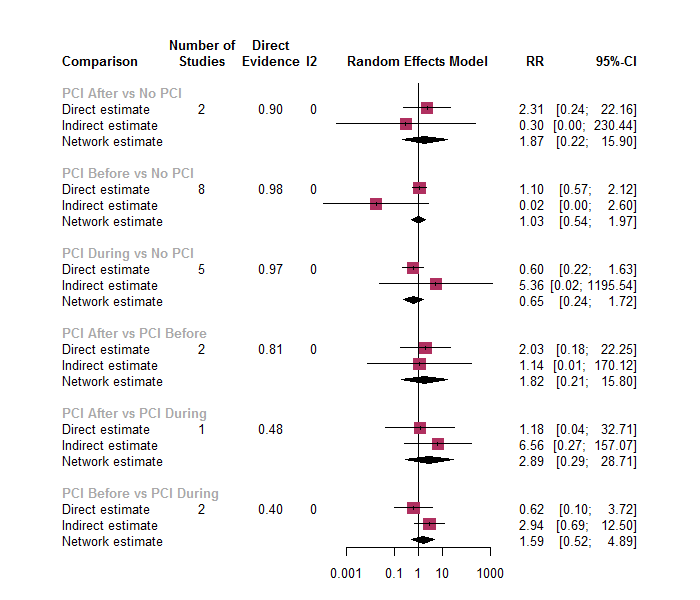

Supplement: Supplementary file 6 — FIGURE S6 Subgroup analysis of 30‐day stroke. [file CLC-47-e24324-s007.png]

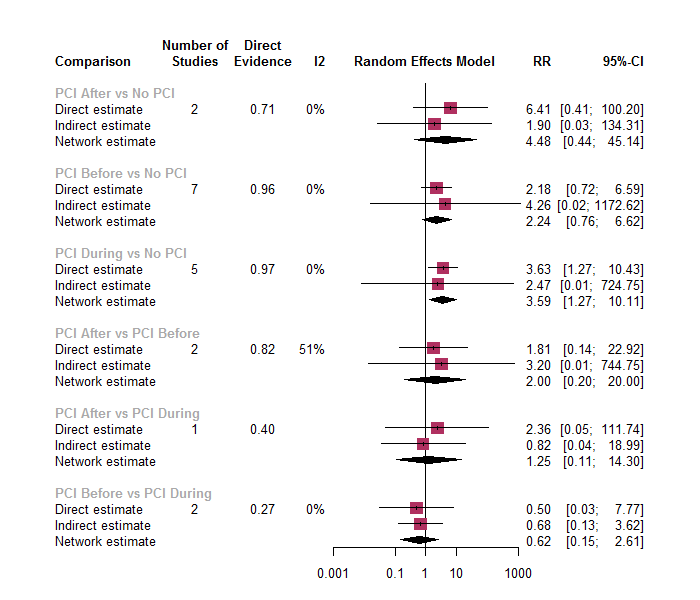

Supplement: Supplementary file 7 — FIGURE S7 Subgroup analysis of 30‐day myocardial infarction. [file CLC-47-e24324-s006.png]

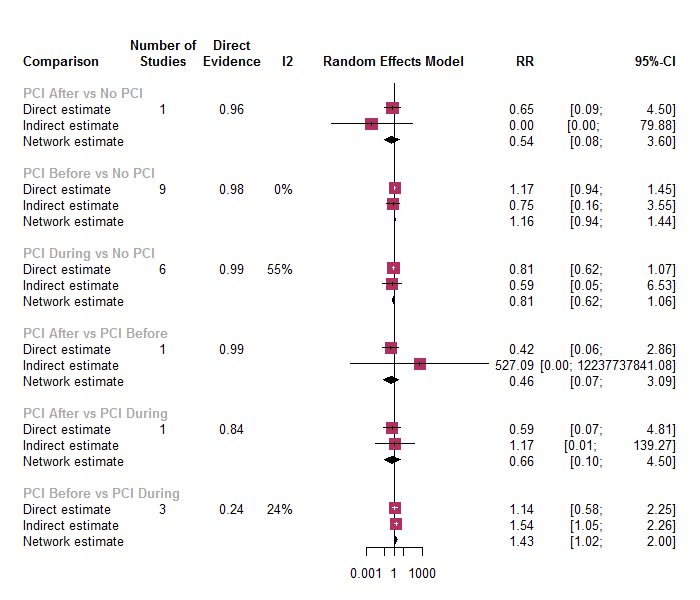

Supplement: Supplementary file 8 — FIGURE S8 Subgroup analysis of 6 months permanent pacemaker placement. [file CLC-47-e24324-s002.png]

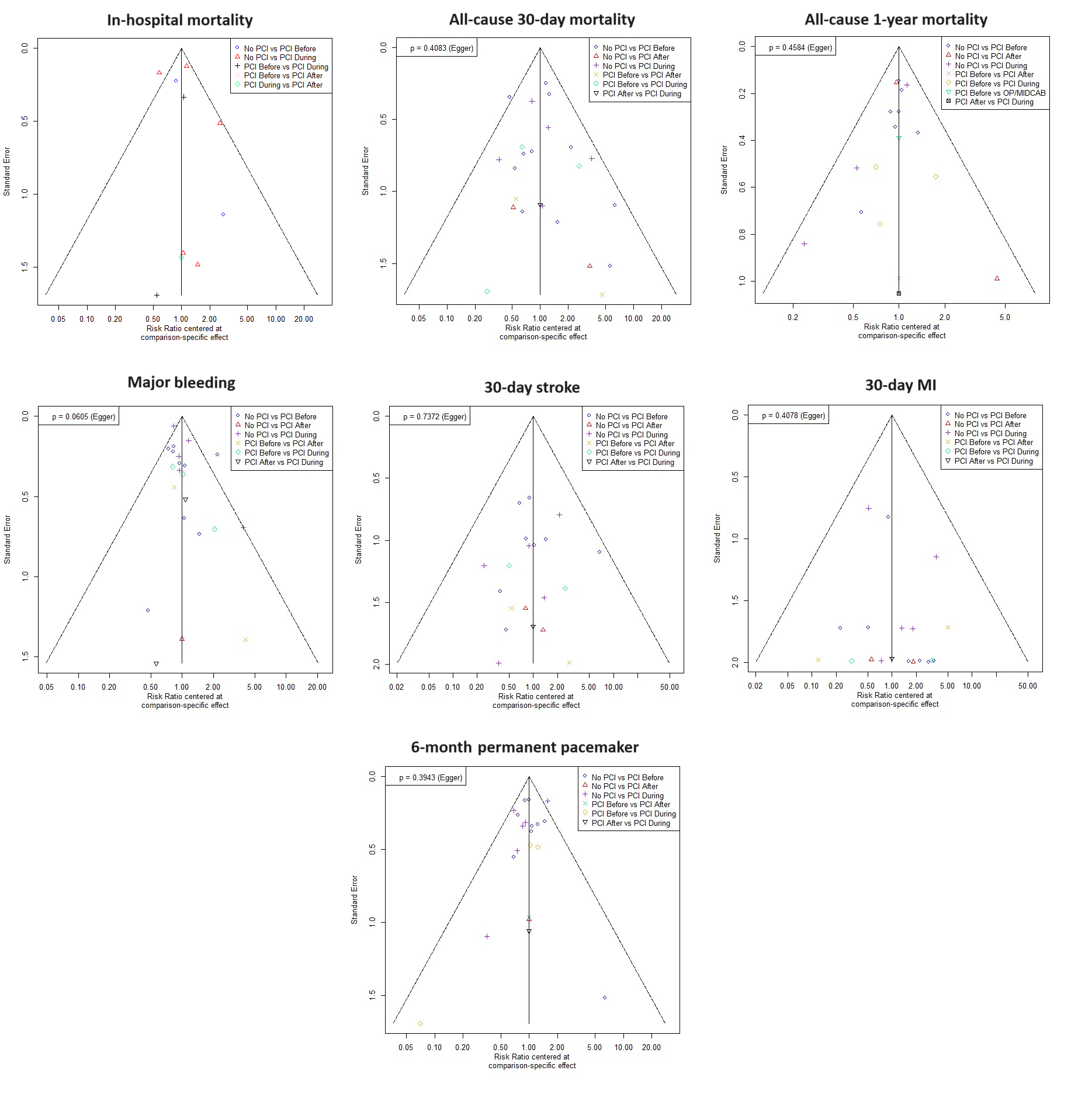

Supplement: Supplementary file 9 — FIGURE S9 Funnel plots representing publication bias. [file CLC-47-e24324-s004.png]
