# Supplementary material for: Comparative Analysis of PCI Strategies in Aortic Stenosis Patients Undergoing TAVI: A Systematic Review and Network Meta‐Analysis
Source: Clin Cardiol. 2024 Jul 26;47(8):e24324. doi: 10.1002/clc.24324 (PMC11272956; doi:10.1002/clc.24324)
Supplement: Supplementary file 10 — Supporting information. [file CLC-47-e24324-s003.docx]

**Table S1.** Excluded articles after full-text screening

| **Title** | **Reason of exclusion** |
| --- | --- |
| 1-Year Clinical Outcomes in Women After Transcatheter Aortic Valve Replacement: Results From the First WIN-TAVI Registry | No PCI |
| A Comparison of Clinical Outcomes According to the Timing of PCI and TAVR: A Pooled Analysis From a Multicenter Registry | Abstract |
| Adequacy of Coronary Revascularization After Transcatheter Aortic Valve Implantation | Editorial |
| Aortic Stenosis and Coronary Artery Disease: Cost of Transcatheter vs Surgical Management. | Abstract |
| Aortic stenosis in high-risk patients presenting coronary artery disease: Conventional or transcatheter strategy? A propensity score analysis | Abstract |
| Appropriate timing of PCI pre-TAVI | Abstract |
| Assessment of functional significance of coronary artery disease of indeterminate severity with fractional flow reserve in patients with aortic stenosis undergoing transcatheter aortic valve implantation | Abstract |
| Balloon Aortic Valvuloplasty Followed by Impella®-Assisted Left Main Coronary Artery Percutaneous Coronary Intervention in Patients With Severe Aortic Stenosis as a Bridge to Transcatheter Aortic Valve Replacement | Case-series |
| Cardiovascular outcomes of concurrent TAVR and PCI compared to TAVR alone using the U.S. national inpatient sample: 2011-2014 | Abstract |
| Catheter versus surgical approach for the management of concomitant aortic stenosis and coronary artery disease: An inverse probability treatment weighting analysis | Full text not found |
| Catheter versus surgical based therapies for the management of concomitant aortic stenosis and coronary artery disease | Abstract |
| Clinical and Technical Characteristics of Coronary Angiography and Percutaneous Coronary Interventions Performed before and after Transcatheter Aortic Valve Replacement with a Balloon-Expandable Valve | No survival analysis / feasibility of PCI |
| Clinical Characteristics and Vascular Complications in Patients Undergoing Transcatheter Aortic Valve Replacement and Percutaneous Coronary Interventions | Abstract |
| Clinical Impact of Pre-Procedural Percutaneous Coronary Intervention in Low- and Intermediate-Risk Transcatheter Aortic Valve Replacement Recipients. | Control is not suitable |
| Clinical outcome in patients undergoing surgical or percutaneous revascularization associated to valve surgery | Abstract |
| Clinical outcomes in surtavi based on revascularization stratification | Abstract |
| Combined cardiac coronary and valvular interventions as a treatment alternative for patients at high surgical risk | Abstract |
| Combined coronary and aortic valvular intervention - A new treatment alternative for patients at high surgical risk | Abstract |
| Combined elective percutaneous coronary intervention and transapical transcatheter aortic valve implantation | Duplication |
| Combined elective percutaneous coronary intervention and transcatheter aortic valve implantation | Duplication |
| Combined Simultaneous TAVI And PCI In High Surgical Risk Patients | Abstract |
| Combined Upper Extremity Complex Percutaneous Coronary Intervention and Transcatheter Aortic Valve Replacement with No Alternative Access | Full text not found |
| Combined transcatheter aortic valve implantation (TAVI) and stenting of the coronary arteries in patients with severe aortic stenosis | Abstract |
| Combined Transcatheter Aortic Valve Implantation and Percutaneous Coronary Intervention - An Alternative to Combined Cardiac Surgery for High-Risk Patients? | Full text not found |
| Comparative Analysis of TAVR with PCI and SAVR with CABG - A Nationwide Inpatient Sample Database | Abstract |
| Comparison of short term impact of percutaneous coronary intervention on mortality in patients with and without severe aortic stenosis: Implications for transcatheter aortic valve implantation | Abstract |
| Complete revascularisation is not a prerequisite for success in current TAVI practice | Complete vs incomplete revascularization/ not relative |
| Complete revascularization is not a prerequisite for success in current transcatheter aortic valve implantation practice | Duplication |
| Coronary angiography and percutaneous coronary intervention after transcatheter aortic valve replacement with medtronic self-expanding prosthesis: Insights from correlations with computer tomography | Feasibility and success of PCI |
| Coronary angiography and percutaneous coronary intervention after transcatheter aortic valve replacement: Feasibility in clinical practice | Feasibility and success of PCI |
| Coronary artery disease and myocardial revascularization in patients undergoing transcatheter aortic valve replacement | Editorial |
| Coronary Artery Disease and Transcatheter Aortic Valve Replacement: Timing and Patient Selection for Coronary Intervention in Patients Planned for TAVR | Book chapter |
| Coronary artery disease significantly impairs long term outcomes after transcatheter aortic valve implantation | Abstract |
| Coronary Catheterization and Percutaneous Interventions After Transcatheter Aortic Valve Implantation | Feasibility and success of PCI |
| Coronary Intervention After Valve-in-Valve TAVR Enabled by Pre-TAVR BASILICA | Case report |
| Coronary procedures in patients treated by TAVI with a self-expanding aortic bioprosthesis-not an easy matter | No survival analysis / feasibility of PCI |
| Coronary revascularisation before transcatheter aortic valve implantation does not impact on outcome | Full text not found |
| Coronary revascularization prior to transcatheter aortic valve implantation: Patient characteristics and influence on outcome | Abstract |
| Coronary rotational atherectomy during TAVI: Immediate and long-term results of a consecutive series | Full text not found |
| Correlation of brain natriuretic peptide levels in patients with severe aortic stenosis undergoing operative valve replacement or percutaneous transcatheter intervention with clinical, echocardiographic, and hemodynamic factors and prognosis | Not relevant |
| Effect of concomitant coronary artery disease on early outcome after transcatheter aortic valve implantation: Results from the German TAVI registry | Abstract |
| Effect of concomitant coronary artery disease on procedural and late outcomes of transcatheter aortic valve implantation | CAD |
| Effect of experience on results of transcatheter aortic valve implantation using a Medtronic CoreValve System | Not relative |
| Effect of previous coronary artery bypass grafting or percutaneous coronary intervention on outcome following transcatheter aortic valve implantation | Full text unavailable |
| Effects of coronary artery disease in patients undergoing transcatheter aortic valve implantation: A study of age- and gender-matched cohorts | CAD |
| Evaluation of coronary disease among patients undergoing transcatheter aortic valve implantation: propensity score matching analysis | CAD |
| Feasibility and outcomes of combined transcatheter aortic valve replacement with other structural heart interventions in a single session: a matched cohort study | LAA occlusion |
| Feasibility and Safety of Impella-Assisted High-Risk PCI Before TAVR in Patients With Severe Aortic Stenosis | Case series/ feasibility and success of PCI |
| First experience with a combined interventional approach to aortic stenosis and concomitant coronary artery disease | Abstract |
| Gender Differences in the Impact of Coronary Artery Disease and Complete Revascularization on Long-Term Outcomes After Transcatheter Aortic Valve Implantation | Not relevant |
| Hybrid Minimally Invasive Approach for Combined Obstructive Coronary Artery Disease and Severe Aortic Stenosis | Not relative |
| Hybrid treatment of aortic stenosis and coronary artery disease by transcatheter aortic valve replacement and percutaneous coronary intervention | Abstract |
| Impact of Annual Hospital percutaneous coronary intervention volume on transcatheter aortic-valve replacement outcomes | Abstract |
| Impact of Coronary Artery Disease and Percutaneous Coronary Intervention on Transcatheter Aortic Valve Implantation | CAD |
| Impact of coronary artery disease on clinical outcomes among patients undergoing TAVI: An age- and sex-matched cohort study | Abstract |
| Impact of coronary artery disease on indications for transcatheter aortic valve implantation and on procedural outcomes | Full text unavailable |
| Impact of extent of coronary artery disease and percutaneous revascularization assessed by the SYNTAX score on outcomes following transcatheter aortic valve replacement | Not relative |
| Impact of Incomplete Coronary Revascularization on Late Ischemic and Bleeding Events after Transcatheter Aortic Valve Replacement | Comparing complete vs incomplete revascularization |
| Impact of New-generation Hybrid Imaging Technology on Radiation Dose during Percutaneous Coronary Interventions and Trans-femoral Aortic Valve Implantations: A comparison with conventional flat-plate angiography | No survival analysis |
| Impact of Percutaneous Coronary Intervention Within 6 Months Prior to Transcatheter Aortic Valve Replacement: A Multicenter Center Study with Central Angiographic Core Laboratory Analysis | Abstract |
| Impact of preprocedural percutaneous coronary revascularisation on outcomes after TAVI | Abstract |
| Impact of pre-procedural PCI on outcome of patients undergoing TAVI | Abstract |
| Impact of pre-procedural percutaneous coronary intervention on outcomes after transcatheter aortic valve implantation | Abstract |
| Impact of residual coronary atherosclerosis on transfemoral transcatheter aortic valve replacement | Complete vs incomplete revascularization/ not relative |
| Impact of residual coronary artery disease on patients undergoing TAVI: A multicenter study | Abstract |
| Impact of revascularisation on clinical outcome in patients undergoing Transcatheter Aortic Valve Implantation: The UK TAVI Registry | Abstract |
| Impact of the presence of coronary artery disease and timing of revascularization on outcomes of patients undergoing trans-catheter aortic valve replacement: Insights from STS/ACC TVT registry | Abstract |
| Impact of coronary artery disease according to Valve Academic Research Consortium outcomes following TAVI | Abstract |
| Impact of coronary artery disease and percutaneous coronary intervention in women undergoing transcatheter aortic valve replacement: From the WIN-TAVI registry. | CAD |
| Impact of coronary artery disease assessed with the SYNTAX score on outcome in patients undergoing transcatheter aortic valve implantation | Abstract |
| Impact of coronary artery disease on management of patients referred for transcatheter aortic valve implantation | Abstract |
| Impact of coronary artery disease on outcomes after transcatheter aortic valve implantation | Abstract |
| Impact of Coronary Artery Disease Severity Assessed With the SYNTAX Score on Outcomes Following Transcatheter Aortic Valve Replacement | Not about PCI outcomes |
| Impact of Revascularization Completeness on Outcomes of Patients with Coronary Artery Disease Undergoing Transcatheter Aortic Valve Replacement | complete vs incomplete revascularization/ not relative |
| Incidence of in-stent restenosis in pre-transcatheter aortic valve replacement percutaneous coronary intervention | Abstract |
| Incidence, feasibility and outcome of PCI after TAVI with a self-expandable prosthesis | Abstract |
| Incomplete Functional Revascularization Is Associated With Adverse Clinical Outcomes After Transcatheter Aortic Valve Implantation | Full text not found |
| Influence of concomitant coronary artery disease on clinical outcomes of patients with severe aortic stenosis undergoing transcatheter aortic valve implantation | Abstract |
| In-hospital and long-term outcomes of percutaneous balloon aortic valvuloplasty with concomitant percutaneous coronary intervention in patients with severe aortic stenosis | BAV |
| In-Hospital and Mid-Term Outcomes of ECMO Support During Coronary, Structural, or Combined Percutaneous Cardiac Intervention in High-Risk Patients - A Single-Center Experience. | Nothing about the outcome of PCI + TAVI |
| In-hospital outcomes after PCI and TAVI versus combined aortic valve replacement and coronary surgery | Full text not found |
| In-hospital outcomes after PCI and TAVI versus combined aortic valve replacement and coronary surgery | Letter to editor |
| In-stent restenosis percutaneous coronary intervention after chimney stenting during transcatheter aortic valve-in-valve implantation | Full text not found |
| Intraoperative and intermediate-term results of an interdisciplinary Transcatheter Aortic Valve Implantation (TAVI)-program | Abstract |
| Intraoperative and intermediate-term results of an interdisciplinary Transcatheter Aortic Valve Implantation (TAVI)-program | conference Abstract |
| Lack of Association Between Percutaneous Coronary Intervention and Transcatheter Aortic Valve Replacement Outcomes in New York Hospitals | Not explaining PCI + TAVI |
| Long-term outcomes after percutaneous coronary intervention of unprotected left main coronary disease with drug-eluting stents | Abstract |
| Long-term results following transcatheter aortic valve replacement (TAVR) | Abstract |
| Long-term risk of unplanned percutaneous coronary intervention after transcatheter aortic valve replacement | Unplanned PCI |
| Management of Coronary Artery Disease and Conduction Abnormalities in Transcatheter Aortic Valve Implantation | Review article |
| Management of coronary artery disease in patients undergoing transcatheter aortic valve implantation | Abstract |
| Management of coronary artery disease in Transcatheter aortic valve Implantation | Abstract |
| New treatment approach: Combined elective coronary artery stenting and transcatheter aortic valve implantation | Abstract |
| Observations from a real-time, iFR-FFR “hybrid approach” in patients with severe aortic stenosis and coronary artery disease undergoing TAVI | Just about angiography not PCI |
| Optimal timing for percutaneous coronary interventions in patients undergoing TAVI | Abstract |
| Outcome of patients with severe aortic stenosis undergoing ad hoc transcatheter aortic valve implantation without invasive pre-evaluation | Just about invasive angiography before TAVI not PCI |
| Outcomes After Transcatheter Aortic Valve Replacement in Patients with Severe Aortic Stenosis and Diastolic Dysfunction | Just a history of PCI as the confounding variable |
| OUTCOMES ASSOCIATED WITH PERCUTANEOUS CORONARY INTERVENTION PRIOR TO TRANSCATHETER AORTIC VALVE IMPLANTATION | Abstract |
| Outcomes following self-expanding transcatheter or surgical aortic valve implantation in patients at low operative risk stratified by need for revascularization | Abstract |
| Outcomes in Patients WithTranscatheter Aortic Valve Replacementand Left Main Stenting | Duplicate |
| Outcomes in Patients WithTranscatheter Aortic Valve Replacementand Left Main Stenting | Duplicate |
| Outcomes in Patients WithTranscatheter Aortic Valve Replacementand Left Main Stenting | Duplicate |
| Outcomes in Patients WithTranscatheter Aortic Valve Replacementand Left Main Stenting | Duplicate |
| Outcomes of Combined Transcatheter Aortic Valve Replacement and Percutaneous Coronary Interventions | Abstract |
| Outcomes of concomitant transcatheter aortic valve replacement and percutaneous coronary intervention in high-risk patients with severe aortic stenosis and obstructive coronary artery disease | Abstract |
| OUTCOMES OF DIABETIC PATIENTS COMPARED TO NON-DIABETIC PATIENTS UNDERGOING PERCUTANEOUS CORONARY INTERVENTION AND SUBSEQUENT TRANSCATHETER AORTIC VALVE REPLACEMENT | Abstract |
| PCI after TAVR—What’s the Price of Reentry? | Editorial |
| Percutaneous Coronary Intervention in Patients With Severe Aortic Stenosis | Not TAVI |
| Percutaneous coronary intervention after self-expanding transcatheter aortic valve replacement | Abstract |
| Percutaneous Coronary Intervention After Transcatheter Aortic Valve Implantation in the Netherlands | Abstract |
| Percutaneous Coronary Intervention and Transcatheter Aortic Valve Replacement Within the Same Admission | Abstract |
| Percutaneous Coronary Intervention Before Transcatheter Aortic Valve Replacement | Abstract |
| Percutaneous Coronary Intervention Before Transcatheter Aortic Valve Replacement: A Propensity Score-Weighted Analysis | Abstract |
| Percutaneous Coronary Intervention Prior to Transcatheter Aortic Valve Replacement: A Matched Cohort Analysis | Abstract |
| Percutaneous coronary ostia catheterization and intervention after Tavi | Abstract |
| Percutaneous vs. surgical treatment strategy in patients with severe aortic stenosis and concomitant coronary artery disease: A 3excluded-day outcome single-centre analysis | Abstract |
| Perivalvular Approach to Percutaneous Coronary Intervention After Transcatheter Aortic Valve Replacement | Case report |
| Physiological Versus Angiographic Guidance for Myocardial Revascularization in Patients Undergoing Transcatheter Aortic Valve Implantation | No report of outcomes in different PCI timings |
| Procoagulant effect of extracellular vesicles in patients after transcatheter aortic valve replacement or transcatheter aortic valve replacement with percutaneous coronary intervention | Not relevant |
| Prognostic impact of coronary lesions and its revascularization in a 5-year follow-up after the TAVI procedure | Full text unavailable |
| Revascularizing coronary artery disease in patients undergoing transcatheter aortic valve implantation | Letter to editor |
| Rotational atherectomy during transcatheter aortic valve implantation | Abstract |
| Routine Screening of Coronary Artery Disease With Computed Tomographic Coronary Angiography in Place of Invasive Coronary Angiography in Patients Undergoing Transcatheter Aortic Valve Replacement | No report of outcomes in different PCI timings |
| Routine revascularization with percutaneous coronary intervention in patients with coronary artery disease undergoing transcatheter aortic valve implantation - the third nordic aortic valve intervention trial - NOTION-3 | RCT design |
| Safety and effectiveness of a selective strategy for coronary artery revascularization before transcatheter aortic valve implantation | CAD |
| Safety and feasibility of rotational atherectomy in octogenarians with severe aortic stenosis and calcific coronary artery disease undergoing evaluation for transcatheter aortic valve replacement: A retrospective observational analysis | Abstract |
| Simple, effective and safe vascular access site closure with the double-ProGlide preclose technique in included62 patients receiving transfemoral transcatheter aortic valve implantation | No PCI |
| Simultaneous multivessel percutaneous coronary intervention and transfemoral transcatheter aortic valve implantation with ACURATE neo. | Case report |
| Simultaneous transcatheter aortic valve replacement and percutaneous coronary intervention-a retrospective study evaluating mortality, incidence of acute kidney injury and radiation exposure | Abstract |
| Successful percutaneous coronary intervention after transcatheter aortic valve implantation with CoreValve bioprosthesis | Case report |
| Surgical aortic valve replacement vs. transcatheter aortic valve implantation in octogenarians | Case report |
| Surgical versus Interventional Treatment of Concomitant Aortic Valve Stenosis and Coronary Artery Disease | Duplicate |
| Surgical vs interventional treatment of aortic stenosis and coronary artery disease | Full text unavailable |
| Technical Characteristics and Feasibility of Coronary Angiography and Percutaneous Coronary Interventions Performed after Transcatheter Aortic Valve Replacement with Self-Expanding Valves | Feasibility |
| The effect of staged PCI followed by TAVI on periprocedural events and long-term mortality outcomes: results from a five-year TAVI registry study | Abstract |
| The effects of pre-existing significant coronary artery disease defined by QCA analysis upon outcome after transcatheter aortic valve implantation using the Edwards bioprosthesis | Abstract |
| The effects of pre-existing significant coronary artery disease defined by QCA analysis upon outcome after transcatheter aortic valve implantation using the Edwards bioprosthesis | Abstract |
| The effects of pre-existing significant coronary artery disease upon outcome after transcatheter aortic valve implantation using the edwards bioprosthesis | Abstract |
| The impact of concomitant percutaneous coronary intervention in high-risk patients with aortic stenosis and coronary artery disease on clinical outcomes in patients undergoing transcatheter aortic valve implantation | Abstract |
| The impact of coronary artery disease on clinical outcomes in patients undergoing transcatheter aortic valve implantation | Abstract |
| The impact of coronary artery disease on survival after TAVI | Abstract |
| The time is always right to do what is right: TAVI and timing of PCI | Editorial |
| Timing of Staged Percutaneous Coronary Intervention Prior to Transcatheter Aortic Valve Implantation: Outcome Implications | Duplicate |
| To PCI or Not: A single center experience on elective PCI Prior to TAVR | Abstract |
| Transcatheter aortic valve implantation | Review article |
| Transcatheter Aortic Valve Implantation in Very High-Risk Patients With EuroSCORE of More Than 4excluded% | Not speaking about PCI |
| Transcatheter aortic valve implantation and concomitant percutaneous coronary intervention in high-risk patients with severe aortic stenosis | Abstract |
| Transcatheter Aortic Valve Implantation in Nonagenarians | Just 7 patients with concomitant PCI without talking about the outcomes |
| Transcatheter aortic valve implantation in patients with and without concomitant coronary artery disease: Comparison of characteristics and early outcome in the German multicenter TAVI registry | Comparing CAD with non CAD (Not reporting outcome of PCI) |
| Transcatheter aortic valve implantation in patients with percutaneous coronary intervention to the left main coronary artery | Abstract |
| Transcatheter aortic valve implantation: The first applications and early results in Turkey | Not English |
| Treatment of coronary artery disease in transcatheter aortic valve implantation patients: Why, when, what to treat? | Abstract |
| Trends in vascular complications and associated treatment strategies following transfemoral transcatheter aortic valve replacement | Just history of PCI |
| Two-stage hybrid coronary intervention and minimally invasive aortic valve replacement for patients with concomitant aortic valve and coronary artery disease | Abstract |
| Two-year outcomes for patients with severe symptomatic aortic stenosis treated with transcatheter aortic valve implantation | Just history of PCI |
| Variation in the Timing of Percutaneous Coronary Intervention and Outcomes in Patients Undergoing Transcatheter Aortic Valve Replacement | Abstract |
| Variation in the Timing of Percutaneous Coronary Intervention and Outcomes in Patients Undergoing Transcatheter Aortic Valve Replacement | Abstract |
| Impact of coronary artery disease in patients undergoing transfemoral transcatheter aortic valve implantation | CAD |
| Impact of concomitant coronary artery disease and percutaneous coronary intervention on procedural and outcomes with severe aortic stenosis performing Transcatheter Aortic Valve Implantation | CAD |
| Feasibility and safety of transfemoral transcatheter aortic valve implantation performed with a percutaneous coronary intervention-like approach | Feasibility and safety of a PCI |
| Utility of routine invasive coronary angiography prior to transcatheter aortic valve replacement | Not relevant |
| Trend, demographics and outcomes of concurrent PCI with TAVR hospitalizations 2excludedincluded2-2excludedincluded8; an analysis from the National Inpatient Sample | Correspondence |
| Outcomes of a hybrid approach of percutaneous coronary intervention followed by minimally invasive aortic valve replacement | No TAVI |
| Impact of coronary artery disease on outcomes of severe aortic stenosis treatment with transcatheter aortic valve implantation | CAD |
| Management of concomitant coronary artery disease in patients undergoing transcatheter aortic valve implantation: The United Kingdom TAVI Registry | Severity of CAD |
| Management of Myocardial Revascularization in Patients With Stable Coronary Artery Disease Undergoing Transcatheter Aortic Valve Implantation | Comparing complete and incomplete revascularization |

**Table S2.** Baseline characteristics of the included articles (Part 2)

| **First author, year** | **Country** | **Transcatheter heart valve** |
| --- | --- | --- |
| Abramowitz (1), 2014 | Israel | CoreValve, Sapien |
| Allali (2), 2016 | Germany | self-expanding MCV, Sapien XT, Sapien 3, Lotus |
| Alperi (3), 2021 | Canada | Sapien, Sapien XT, CoreValve |
| Barbanti (4), 2017 | Italy | Sapien XT, Sapien 3, CoreValve, Evolut R, Acurate neo, Portico |
| Baumbac (5), 2019 | Germany | - |
| Benseba (6), 2023 | Canada | Sapien XT, Sapien 3, sapien ultra, CoreValve, Evolut R, Evolut PRO |
| Beohar (7), 2022 | USA | - |
| Chakravarty (8), 2016 | USA | Sapien, CoreValve,  Direct Flow |
| Conradi (9), 2011 | Germany | Sapien, CoreValve |
| Faroux (10), 2020 | Canada | balloon-expandable, self-expanding, mechanically expandable |
| Ghrair (11), 2020 | USA | - |
| Griese (12), 2014 | Germany | Sapien, Sapien XT, CoreValve, Acurate |
| Huczek (13), 2014 | Poland | balloon- and self-expandable aortic valve prosthesis of  the first and second generation |
| Kaihara (14), 2021 | Japan | CoreValve, Sapien XT |
| Karaduman (15), 2021 | Turkey | Sapien XT, Sapien 3, Lotus |
| Kneizeh (16), 2022 | Germany | - |
| Kodra (17), 2022 | USA | - |
| Kumar (18), 2020 | Georgia | Sapien, Sapien XT, Sapien 3, CoreValve, Lotus, Evolut R, Evolut Pro, Direct Flow |
| Ochiai (19), 2020 | Japan | Sapien, Sapien-XT, Sapien 3, CoreValve, Evolut R, Evolut PRO |
| Pasic (20), 2012 | Germany | Sapien |
| Patterson (21), 2021 | UK | Sapien, Sapien XT, CoreValve, CoreValve Evolut, CoreValve Evolut R, Evolut Pro, Lotus, Symetis |
| Penkalla (22), 2014 | Germany | Sapien |
| Rheude (23), 2023 | Germany | S3/S3 Ultra, SXT, Evolut R/PRO, CoreValve, Portico, Lotus, Acurate neo/neo2, Allegra |
| Santana (24), 2017 | USA | - |
| Shah (25), 2023 | USA | - |
| Søndergaard (26), 2018 | Denmark | CoreValve |
| Tarantini (27), 2020 |  | Sapien 3 |
| Tran (28), 2022 | USA | - |
| Valvo (29), 2023 | Italy | Sapien 3, Sapien XT, Evolut R/PRO, CoreValve, Portico, Lotus, Acurate Neo |
| van Rosendael (30), 2015 | Netherlands | - |
| Wenaweser (31), 2011 | Switzerland | CoreValve, Sapien |
| Zivelonghi (32), 2017 | Italy | Sapien, CoreValve, Lotus |
